# Deregulation of Annexin-A1 and Galectin-1 Expression in Precancerous Gastric Lesions: Intestinal Metaplasia and Gastric Ulcer

**DOI:** 10.1155/2014/478138

**Published:** 2014-02-25

**Authors:** Ana Flávia Teixeira Rossi, Márcia Cristina Duarte, Ayla Blanco Poltronieri, Marina Curado Valsechi, Yvana Cristina Jorge, Dalísio de-Santi Neto, Paula Rahal, Sonia Maria Oliani, Ana Elizabete Silva

**Affiliations:** ^1^Department of Biology, São Paulo State University (UNESP), Câmpus São José do Rio Preto, Rua Cristóvão Colombo 2265, 15054-000 São José do Rio Preto, SP, Brazil; ^2^Legal Medicine Department and Pathology Service, Hospital de Base, Avenida Brigadeiro Faria Lima 5544, 15090-000 São José do Rio Preto, SP, Brazil

## Abstract

*Objective.* Annexin-A1 (*ANXA1*/AnxA1) and galectin-1 (*LGALS1*/Gal-1) are mediators that play an important role in the inflammatory response and are also associated with carcinogenesis. We investigated mRNA and protein expression in precancerous gastric lesions that participate in the progression cascade to gastric cancer, such as intestinal metaplasia (IM) and gastric ulcer (GU). *Methods.* Quantitative real-time PCR (qPCR) and immunohistochemical techniques were used to analyze the relative quantification levels (RQ) of *ANXA1* and *LGALS1* mRNA and protein expression, respectively. *Results.* Increased relative expression levels of *ANXA1* were found in 100% of cases, both in IM (mean RQ = 6.22 ± 0.06) and in GU (mean RQ = 6.69 ± 0.10). However, the *LGALS1* presented basal expression in both groups (IM: mean RQ = 0.35 ± 0.07; GU: mean RQ = 0.69 ± 0.09). Immunohistochemistry revealed significant positive staining for both the AnxA1 and Gal-1 proteins in the epithelial nucleus and cytoplasm as well as in the stroma of the IM and GU groups (*P* < 0.05) but absence or low immunorectivity in normal mucosa. *Conclusion*. Our results bring an important contribution by evidencing that both the AnxA1 and Gal-1 anti-inflammatory proteins are deregulated in precancerous gastric lesions, suggesting their involvement in the early stages of gastric carcinogenesis, possibly due to an inflammatory process in the gastric mucosa.

## 1. Introduction 

Precancerous lesions are related to the development of tumors in several organs, such as intestinal-type gastric cancer that develops through the progression of various sequential lesions that frequently start with a *Helicobacter pylori *infection. This infection causes superficial gastritis that can progress to chronic atrophic gastritis, intestinal metaplasia, dysplasia, and, finally, carcinoma [1]. Intestinal metaplasia, characterized by the differentiation of gastric stem cells into intestinal-phenotype cells [2], is associated with more than 80% of intestinal-type adenocarcinoma [3]. Besides this metaplasia-dysplasia-carcinoma pathway, gastric carcinogenesis can originate from gastric ulcer [4], a lesion in the mucosa that develops in low acid concentration sites and severe inflammation [5, 6]. Eighty-five percent of gastric ulcer cases occur in the presence of *H. pylori *infection [5, 7].

The progression of the lesions cascade depends on many genetic factors in both the host and the bacterium, besides environmental factors [8]. *H. pylori* may persist for many years in the host, causing a chronic inflammation. This results in a great amount of inflammatory mediators and the reactive oxygen and nitrogen species that induce genetic and epigenetic changes in protooncogene and tumor suppressor genes, influencing the emergence of cancer [9]. Such bacteria populations are heterogeneous and contain virulence factors such as *cagA (cytotoxin-associated gene A antigen),* which produces a protein that acts in many cellular events such as cytoskeleton rearrangement, cellular polarity breaking, and mitogenic and proapoptotic responses [10]. This virulence genotype, however, is not found in all the strains; its occurrence has a relation with major gastric mucosa inflammation [11] and high risk of gastric cancer development [12].

Outstanding among the inflammatory mediators that activate the immune response cascade are the anti-inflammatory proteins annexin-A1 (AnxA1) and galectin-1 (Gal-1), involved in various inflammatory processes [13, 14]. AnxA1 belongs to a protein superfamily characterized by binding to cellular membranes in a calcium-dependent manner, acting on several cellular and molecular processes [13]. Throughout the inflammatory process, this protein externalizes in the plasmatic membrane, blocking the interaction between leukocytes and endothelium in order to stop the inflammatory cells transmigration to the damaged site [15]. Its anti-inflammatory action is also related to the induction of neutrophil apoptosis [16] and inhibition of phospholipase A2 (PLA2) [17]. Furthermore, it also has a connection to cellular differentiation mechanisms, growth, signal transduction, and cytoskeleton formation [18]. Thus, changes in their expression and subcellular location can contribute to inflammatory diseases and cancer [19–22].

Galectin-1 belongs to a family of *β*-galactoside-binding protein [23]. It has many cellular functions, such as apoptosis [24], cellular signaling, adhesion, migration [25], and proliferation [26], and its action depends on its cellular concentration and location [14]. Its anti-inflammatory function relates to the induction of apoptosis of T-activated cells [24, 27] and the decreased production of proinflammatory cytokines [28, 29]. Furthermore, galectin-1 promotes differentiation of Treg cells controlling their immunosuppressive mechanisms [30, 31] and suppresses dendritic cells maturation favoring a tolerogenic microenvironment [32]. In the tumor microenvironment, the presence of Gal-1 contributes to immunosuppression [31], angiogenesis promotion [33], metastasis [34], and cell transformation [35]. Several types of cancers show changed expression of this anti-inflammatory mediator [36, 37], associated with the increase of cellular proliferation [36] and metastasis [38].

Studies on annexin-A1 and galectin-1 in gastric cancer and precancerous lesions are few and reveal contradictory results. Some of them found AnxA1 and Gal-1 downregulation [39–41], while others reported increase of these proteins in the lesions and tumor tissue [42–46].

Considering that gastric cancer has a high incidence and poor prognosis when diagnosed at a late stage [47], the study of precancerous lesions can provide important information about the initial phases of gastric carcinogenesis, thereby contributing to prevention strategies which may lead to a decrease in its incidence through the identification of potential biomarkers. Recently, we showed a similarly increased expression of annexin-A1 and galectin-1 in chronic gastritis and in gastric cancer, evidencing deregulation in the expression of these proteins during the initial step of gastric carcinogenesis [46]. Based on this finding, we decided to investigate the expression and location pattern of both proteins in other precancerous gastric lesions that participate in the progression cascade to gastric cancer.

In this study, we evaluated the quantitative mRNA expression of genes *ANXA1* and *LGALS1 *and the expression of both proteins in intestinal metaplasia and gastric ulcer lesions. In addition, we also investigated the occurrence of association between the expression levels and infection by *H. pylori *and its *cagA* virulence genotype, besides other risk factors associated with gastric carcinogenesis.

## 2. Materials and Methods

### 2.1. Samples

This research was approved by the local Research Ethics Committee (CEP-IBILCE/UNESP, number 059/11), and written informed consent was obtained from all participants, who also filled out a questionnaire from which we obtained family and occupational data, besides information on smoking and drinking habits.

DNA and cDNA samples of gastric biopsies (antrum and corpus) of the lesion area and adjacent normal mucosa stored in our laboratory from a previous study [48] were used. All subjects were recruited from the Service of Endoscopy, Hospital de Base, São José do Rio Preto, SP, Brazil. A total of 75 samples were evaluated, 36 of which of intestinal metaplasia of individuals without gastric cancer (IM—19 women, 17 men; mean age: 60.53 ± 12.21 years), 29 of gastric ulcer (GU—8 women, 21 men; mean age: 54.62 ± 12.42 years), and 10 from individuals with histologically normal gastric mucosa without dyspepsia, used as controls (C—3 women, 7 men; mean age: 34.20 ± 9.73 years). Subjects with prepyloric or nonsteroid anti-inflammatory drug-induced ulcers were excluded from this study [48].

### 2.2. Molecular Diagnosis for *H. pylori *and *cagA *Genotype

DNA samples of normal mucosa adjacent to gastric lesions were subjected to multiplex PCR containing primers for bacterial genes *UreA* and *tsaA* and for the human constitutive gene *CYP1A1*, to verify DNA integrity and efficiency of the PCR reaction (Table 1). We used 1X buffer, 0.15 *μ*M of each deoxyribonucleotide, 2 mM MgCl_2_, 0.6 *μ*M of each primer, 100 ng genomic DNA, and 1.8 U Platinum* Taq* DNA Polymerase (Invitrogen) in a final volume of 25 *μ*L. The reaction was processed in an automatic thermocycler, with denaturation performed at 94°C for 3 minutes, followed by 35 cycles at 94°C for 45 seconds, 60°C for 30 seconds, and 72°C for 1 minute, and a final extension of 10 minutes at 72°C. The reaction products were subjected to electrophoresis on 3.0% agarose gel stained with ethidium bromide.

The *H. pylori*-positive samples were subjected to a second PCR assay, to investigate the *cagA* virulence genotype. The reaction solution contained 1X buffer, 0.1 *μ*M of each deoxyribonucleotide, 2 mM MgCl_2_, 0.6 *μ*M of each primer (Table 1), 300 ng genomic DNA, and 1 U *Taq* DNA Polymerase (Invitrogen). The reaction conditions were 94°C for 5 minutes for denaturation, 40 cycles of 1 minute at 94°C, 1 minute at 56°C and 1 minute at 72°C, and a final extension of 7 minutes at 72°C. The reaction products were subjected to electrophoresis on 1.0% agarose gel. We used positive and negative controls in all experiments.

### 2.3. Relative mRNA Quantification by Quantitative PCR (qPCR)

Relative quantification of mRNA was performed in an ABI Prism 7300 Sequence Detection System (Applied Biosystems, California, USA), using the SybrGreen PCR Core Reagent (Applied Biosystems, California, USA) protocol and specific primers for the genes *ANXA1, LGALS1,* and *ACTB, *as described in previous studies [46]. Gene *ACTB *was used as endogenous control (reference gene) because it had shown the lowest variation amplification in gastric tissue [48]. Beyond the IM and GU groups, 10 samples of normal gastric mucosa were mixed and submitted to quantitative PCR and used as a calibrator for analysis (standard sample).

Relative quantification (RQ) of genes *ANXA1 *and* LGALS1 *was performed according to the model proposed by Pfaffl [49], compared with pool of samples from normal gastric mucosa and normalized to the *ACTB* reference, according to the formula *R* = (*E*  target)^ΔCt  target  (control−sample)^/(*E*  endogenous)^ΔCt  endogenous  (control−sample)^. RQ was expressed as Log2 mean ± SD, and cases with a value of RQ > 2 were considered upregulated.

### 2.4. Immunohistochemistry

Immunohistochemical analysis was performed on 25 samples of IM, 10 of GU, and 6 of normal mucosa. The GU group samples included lesion, peripheral, healing, and regeneration areas. After deparaffinization, histological sections were submitted to antigen retrieval in citrate buffer and blocking endogenous peroxide activity. The sections were incubated overnight at 4°C with the primary rabbit polyclonal anti-ANXA1 and anti-Gal-1 antibodies (Zymed Laboratories, Cambridge, UK) diluted to 1 : 1000 and 1 : 500, respectively, in 1% bovine serum albumin (BSA in phosphate-buffered saline (PBS)). After incubation with a secondary biotinylated antibody (Dako, Cambridge, UK), staining was detected using a peroxidase-conjugated streptavidin complex, revealed with 3,3′-diaminobenzidine substrate (Dako, Cambridge, UK), and counterstained with hematoxylin. All experiments were carried out with a negative control consisting of a tissue section incubated with PBS and 1% BSA instead of the primary antibody, with all other steps kept equal, confirming that the staining is specific.

Immunostaining was evaluated in stroma, epithelial nuclei, and cytoplasm by densitometric analysis using an arbitrary scale from 0 to 255, performed with AxioVision software under a Zeiss-Axioskop II light microscope. Sixty points, equally distributed in each one of the regions, were scored, and the resulting values were expressed as mean ± SE.

### 2.5. Statistical Analysis

Fisher's exact test was used to determine if there were any significant differences among the groups regarding the presence of bacteria and the virulence of genotype *cagA. *To determine whether an association exists between groups and relative gene expression, we used the unpaired *t*-test with Welch's correction. The same test was used to investigate the existence of an association of risk factors (age, gender, smoking, and drinking) and *H. pylori *infection with the values of relative gene expression. The nonparametric Mann-Whitney test was used to determine whether there was a correlation between the virulence of genotype *cagA *and the relative gene expression. For protein expression, the mean values obtained by densitometry for each region were compared in groups C, IM, and GU, using the ANOVA followed by the Bonferroni test. To determine the association between *H. pylori *infection and the values of protein expression we used the nonparametric Mann-Whitney test. A value was considered significant if *P* < 0.05. 

## 3. Results

### 3.1. Molecular Diagnosis for *H. pylori *and *cagA* Genotype

Forty-six percent (29/63) of all gastric lesion samples were found to be positive for *H. pylori*; 38.9% (14/36) of them belonged to the IM group and 55.5% (15/27) to the GU group. Among them, 75.9% (22/29) were also positive for the *cagA* genotype, 85.7% (12/14) belonging to the IM group and 66.7% (10/15) to the GU group. No significant difference regarding the bacterium infection (*P* = 0.21) or the presence of the *cagA *genotype (*P* = 0.39) was found between the IM and GU groups. All 10 samples of normal mucosa were confirmed by molecular testing as *H. pylori* negative.

### 3.2. Relative Expression of mRNA Determined by q-PCR

**Figure 1:**
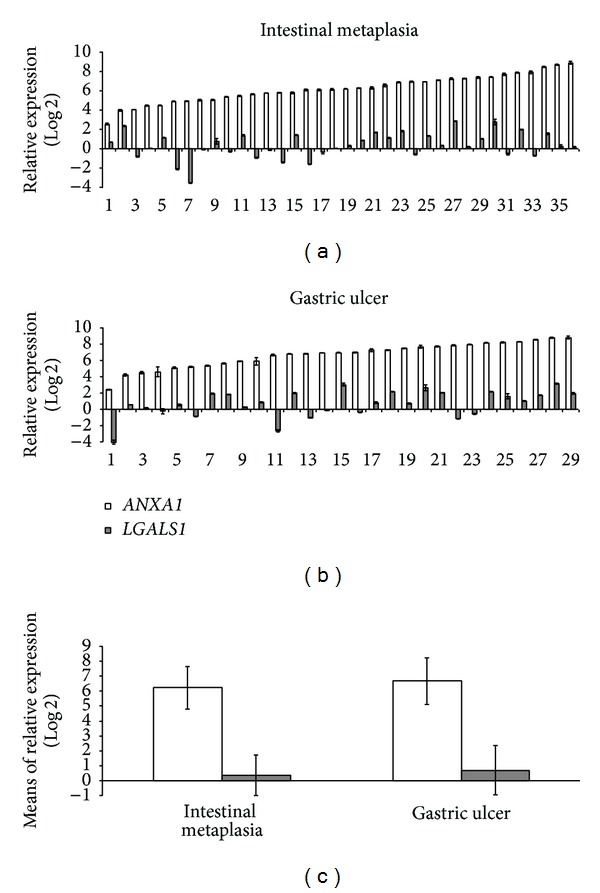
Distribution of relative gene expression (Log2RQ) of *ANXA1 *and *LGALS1 *(a and b) and mean values of relative gene expression in intestinal metaplasia and gastric ulcer groups (c).

The relative gene expression of *ANXA1 *was upregulated in 100% of both the IM (mean RQ = 6.22 ± 1.43) and the GU cases (mean RQ = 6.69 ± 1.56). However, for *LGALS1, *both groups showed basal gene expression, with overexpression in only 36.1% (13/36) of the IM cases (mean RQ = 0.35 ± 1.37) and 44.8% (13/29) of the GU cases (mean RQ = 0.69 ± 1.65) (Figure 1 and Table 2). There was no significant difference between the IM and GU groups regarding the mean gene expression values for either the *ANXA1 *or the *LGALS1 *gene (*P* = 0.21 and *P* = 0.38, resp.).

We also evaluated a possible association among the relative expression levels of *ANXA1* and *LGALS1* mRNA and the risk factors age, gender, smoking, drinking, *H. pylori *infection, and *cagA *genotype (Table 2). We found a significant increase only in the mean level of *LGALS1 *mRNA of the women (0.80 ± 1.30) compared to the men (−0.15 ± 1.29) in the IM group (*P* = 0.03).

### 3.3. AnxA1 and Gal-1 Expressions in Precancerous Gastric Lesions

**Figure 2:**
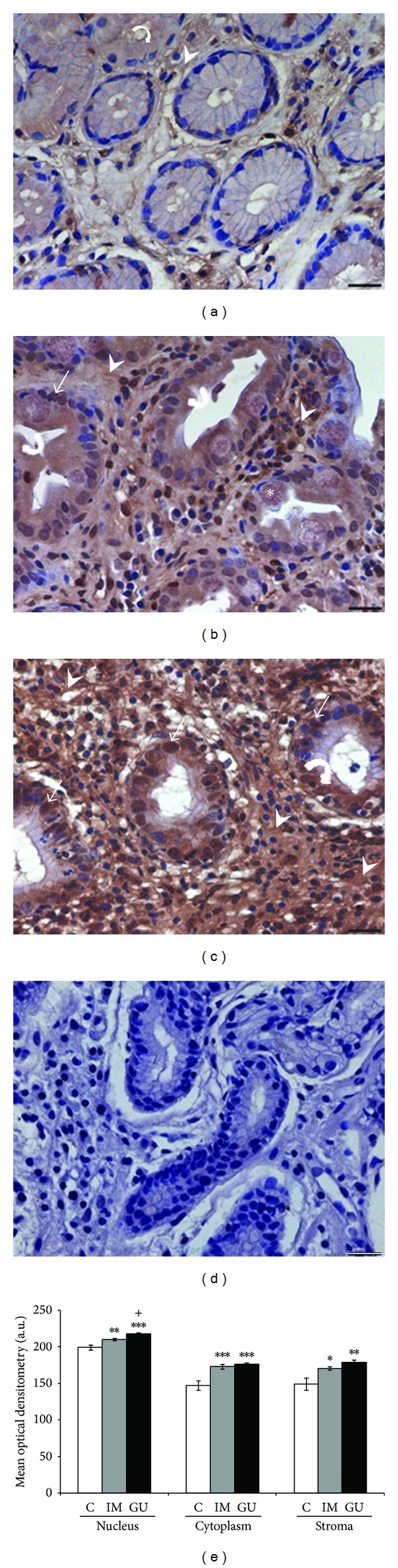
Expression of annexin-A1 protein in gastric mucosa (a–c). (a) Expression of AnxA1 in normal mucosa (C), predominantly in the cytoplasm of epithelial cells (curved arrow) and stroma (arrowhead). (b) Increased expression after intestinal metaplasia (IM) in the nucleus (arrow), cytoplasm (curved arrow), and stromal region (arrowhead). (c) Higher AnxA1 expression can be observed in peripheral area of gastric ulcer (GU) especially in stroma (arrowhead) and epithelial cells (curved arrow and arrow). (d) Absence of immunoreactivity in the reaction control. Asterisk: goblet cell. Hematoxylin of Harris counterstain. Bar: 20 *μ*m. (e) Densitometry analyses (mean ± SE). *P in relation to C (*P < 0.05; **P < 0.01; ***P < 0.001); ^+^ *P* < 0.05 in relation to IM. a.u.: arbitrary unit.

**Figure 3:**
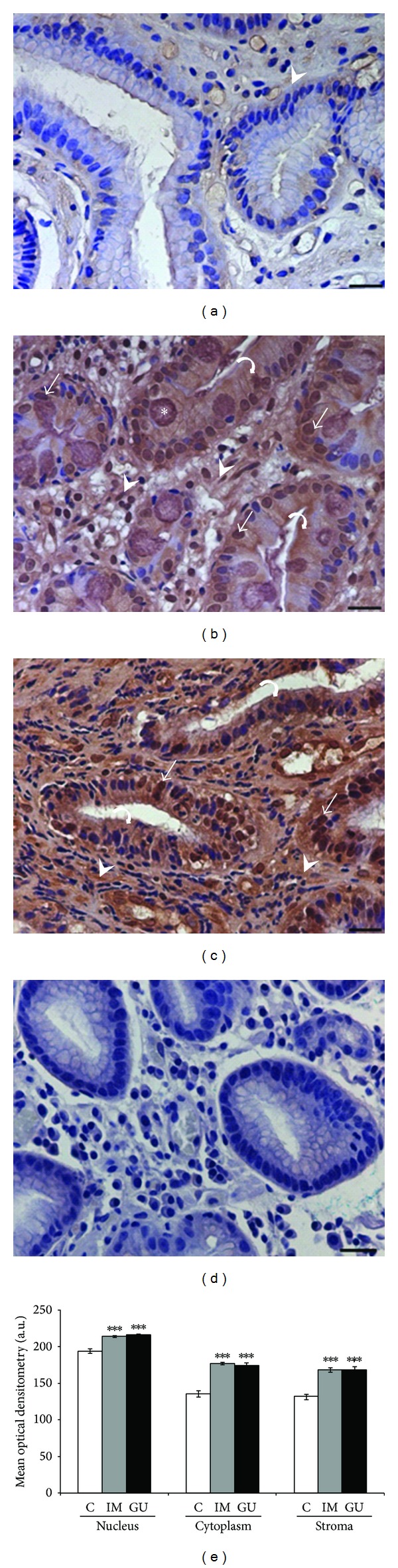
Expression of galectin-1 protein in the gastric mucosa (a–c). (a) Expression of Gal-1 in normal mucosa (C) in the stroma (arrowhead). (b) Intense immunostaining was observed in intestinal metaplasia (IM) in the nucleus (arrow), cytoplasm (curved arrow), and stromal region (arrowhead). (c) Peripheral area of gastric ulcer (GU) with high expression in stroma (arrowhead) and epithelial cells (curved arrow and arrow). (d) Absence of immunoreactivity in the reaction control. Asterisk: goblet cell. Hematoxylin of Harris counterstain. Bar: 20 *μ*m. (e) Densitometry analyses (mean ± SE). *P in relation to C (***P < 0.001). a.u.: arbitrary unit.

In normal mucosa, the AnxA1 and Gal-1 proteins showed low expression in the stroma, while in the epithelium there was no immunostaining for Gal-1, and AnxA1 presented low immunoreactivity (Figures 2(a) and 3(a)). In the IM and GU groups, both proteins exhibited high immunostaining in the epithelial nuclei and cytoplasm as well as in the stroma. For Gal-1, these groups showed high staining throughout the extension of the epithelial cytoplasm (Figures 3(b) and 3(c)). However, regarding the AnxA1 protein, the GU samples presented higher immunostaining in the basal portion of the epithelial cytoplasm (Figure 2(c)), while the IM group showed immunoreactivity in the whole cytoplasm (Figure 2(b)). The specificity of the reactions was confirmed by negative controls (Figures 2(d) and 3(d)).

The mean optical densitometry values for AnxA1 ranged from 147.5 to 218.2 in the three regions analyzed (epithelial nucleus, cytoplasm, and stroma) and, for Gal-1, from 131.6 to 215.8. There was a significant difference in the expression of both proteins in the epithelial cytoplasm and nuclei and in the stroma of the IM and GU groups compared to the control group (*P* < 0.05). Moreover, we found a significant difference (*P* < 0.05) between the IM and GU groups regarding the nuclear expression of the AnxA1 protein in the gastric epithelium (Figures 2(e) and 3(e)).

## 4. Discussion

This is the first study that revealed increased annexin-A1 expression in human intestinal metaplasia and gastric ulcer and increased galectin-1 expression in gastric ulcer, two precancerous gastric lesions, indicating that these anti-inflammatory mediators can exert effects on the initial steps of stomach carcinogenesis. Our results also demonstrated the location of these proteins in the affected tissue and showed that gene expression alterations occur regardless of *H. pylori *infection and *cagA* virulence genotype.

The relative gene expression of *ANXA1* was 6.2- and 6.7-fold increased, respectively, in intestinal metaplasia and gastric ulcer and these results were confirmed by AXNA1 protein expression analysis. Regarding *LGALS1, *the relative gene expression presented basal values in both lesions. However, the protein expression was significantly higher in both intestinal metaplasia and gastric ulcer compared to normal mucosa. In the present study, the qPCR and immunohistochemistry techniques performed are reliable due to the absence of quantification and immunostaining, respectively, in the negative controls. Several studies have shown minimal or limited correlation between mRNA and protein levels, particularly when using average expression values [50, 51]. This is justified by the existence of posttranscriptional processes such as translation, posttranslational mechanisms, and degradation, which influence the protein abundance in a specific tissue [50–52]. Therefore, mRNA expression levels do not always predict corresponding protein levels [52].

The increase of annexin-A1 expression in the lesions analyzed indicates a possible involvement in the progression of gastric carcinogenesis from early lesions, as observed in our previous study in chronic gastritis [46] and now in intestinal metaplasia and gastric ulcer, to gastric cancer. The role of this protein in carcinogenesis is still uncertain, and its effect may seem tissue specific [22] and dependent on its subcellular location [53]. Particularly in gastric cancer, the few studies show conflicting results, some of them revealing overexpression of AnxA1 [44, 46, 53] and others downregulation [39, 41]. Regarding precursor lesions, Martin et al. [45] observed increased expression of this protein in healing areas of gastric ulcer induced in rats, but we did not find any study in intestinal metaplasia. Interestingly, other studies in cancers that originate from multistep processes also show alterations in the AnxA1 expression, both in precursor lesions and carcinoma, indicating a greater proximity between precancerous and cancerous stages [19, 21]. For example, in oral squamous cell carcinoma, this protein presented decreased expression in the plasmatic membrane of tumor cells and premalignant lesions compared to normal oral mucosa [21]. Similarly, Alves et al. [19] also observed reduced expression of AnxA1 in premalignant lesions diagnosed as oral leukoplakia and in laryngeal squamous cell carcinoma.

The molecular pathway of gene *ANXA1* in the modulation of carcinogenesis is related to its action as a substrate to the epidermal growth factor receptor (EGFR) and protein kinase C (PKC), which implies its involvement in signal transduction pathways to cancer [54, 55]. The AnxA1 expression influences the mitogen-activated protein kinases (MAPKs) pathway that is associated with the regulation of biological functions such as cell proliferation, differentiation, and apoptosis [56]. However, its way of acting on these pathway members remains uncertain. Increased* ANXA1 *expression was found to be associated with constitutive expression of extracellular signal-regulated kinases- (ERK-)1 and 2 in macrophages [54] and vascular smooth muscle cells, contributing to the reduction in the cell proliferation rate through downregulation of cyclin D1 [57]. Yet, in prostate cancer, the involvement of AnxA1 in this pathway was not by an antiproliferative but rather a proapoptotic action through p38 and JNK (*c-Jun N-terminal kinase*) activation [56]. Thus, the action of AnxA1 on proliferation seems to depend on the tissue type. An antiproliferative activity was found in lung adenocarcinoma [58], macrophages, and smooth muscle tissue [57]. On the other hand, proliferation stimulation was observed in hepatocytes, in which AnxA1 was related to EGF [59], and in breast cancer; in the latter, it was associated with formyl peptide receptor (FPR2) binding and increased levels of cyclin D1 [60]. In gastric cancer patients, the high expression of this protein was related to the promotion of invasiveness and shorter survival, and this relationship occurred through the FPR/ERK/ITGB1BP1 pathway [53].

The Gal-1 protein shows increased expression in various types of cancer and is associated in most of the cases with aggressiveness and metastatic potential [36, 37, 61–63]. In gastric cancer, however, Bektas et al. [40] did not find expression of this protein in most of the cases studied, whereas Jorge et al. [46] observed increased expression of Gal-1 in the stroma and epithelium of cancerous gastric mucosa. Chen et al. [43] observed increased expression of the *LGALS1* mRNA in the stomach cell line TMC-1, suggesting that this protein is important in the metastasis process. Another *in vitro* study showed that the tumor suppressor gene *RASSF1A* positively regulates the *LGALS1* mRNA level, leading to the suppression of the NF-kB signaling pathway, which indicates that Gal-1 expression may be related to cell cycle arrest [64]. Regarding gastric lesions, we previously observed elevated protein expression in chronic gastritis [46], but Bektas et al. [40] did not detect any increased expression in tumor-associated metaplasia and dysplasia, and we found no reports about this finding in gastric ulcer. However, in the present study, Gal-1 presented increased expression in both intestinal metaplasia not associated with cancer and gastric ulcer. In contrast, in colorectal adenoma, a precancerous lesion of the colon presented downregulation of Gal-1 compared to normal mucosa, showing a change in gene expression in early stages of colorectal carcinogenesis [65].

Galectin-1 has many functions involving carcinogenesis. High expression of this protein in the tumor microenvironment contributes to the development and progression of the tumor by promoting environmental immunosuppression, angiogenesis, and metastasis [30]. Gal-1 induces Fas-mediated apoptosis in immature thymocytes and activated T lymphocytes [66], resulting in the activation of caspase 8 and increased mitochondrial membrane potential [27]; in lymphoblastoid Jurkat cells, the apoptosis triggered by Gal-1 occurs via JNK/c-Jun/AP-1 (activating protein-1 transcription factor) [24]. Thus, Gal-1 contributes to conferring immune privilege to tumors through apoptosis of T cells. Gal-1 released in the tumor microenvironment also acts on activated endothelial cells, promoting *H-Ras* signaling via the Raf/MAPK/MEK/ERK pathway, which results in the proliferation and migration of endothelial cells and consequent generation of new vessels [67]. Taken together, these data, combined with the results of the present study, indicate that galectin-1 can also influence the gastric carcinogenesis process, contributing to the progression of the lesions cascade from the initial stages.

Besides the change in expression levels, the location of these anti-inflammatory proteins may play an important role in the development of different pathological conditions [19]. We analyzed the location of both proteins in the premalignant lesions, which presented increased expression in relation to normal mucosa in the stroma and in the epithelial cytoplasm and nucleus. AnxA1 is normally detected in the cytoplasm of many tissues [20]. Exposure to hydrogen peroxide, heat, arsenic, and EGF promotes its translocation to the nucleus [68]. In gastric cancer, nuclear expression of this protein was found to be associated with advanced disease and poor prognosis [41], and in oral carcinoma it is a predictor of lower survival [20]. However, we cannot affirm how significant the nuclear expression of this protein was in the precancerous lesions studied here. Differently, Gal-1 has both cytoplasmic expressions as nuclear [63], while stromal expression is associated with differentiation stage and metastasis in gastric cancer [40]. Our results showed significantly higher expression of this protein in the stroma of intestinal metaplasia and gastric ulcer samples compared to normal mucosa, which may contribute, as shown by Hittelet et al. [69] in colon cancer, to the progression from a precancerous to a cancerous stage.

When we analyzed the association of *ANXA1* and* LGALS1 *mRNA expression levels with the risk factors in the two lesions studied, we only found an association between the relative expression of *LGALS1* and gender in the IM group, with women showing higher expression than men (RQ = 0.80 *versus* −0.15). We did not find any association between the expression levels of both genes and *H. pylori* infection, nor with the *cagA *genotype. Nevertheless, considering the important role of bacterial infection in the development and progression of gastric cancer, investigations in larger populations are needed to confirm these results.

Regarding the role of the higher expression of galectin-1 in women, Von Wolff et al. [70] reported elevated expression of this protein in human endometrium during the menstrual cycles and in maternal deciduas, suggesting a role of this protein in maintaining pregnancy. Other studies suggest that sex steroids may regulate *LGALS1* expression in female reproductive tissues of humans and rats [70, 71]. Choe et al. [71] had observed increased expression of this gene in the uterus of ovariectomized rats six hours after treatment with 17*β*-estradiol and 12 hours after inoculation of progesterone, the effects being blocked by antagonists of these hormones. It has therefore been suggested that estrogen and progesterone receptors are involved in galectin-1 expression [72], so the higher *LGALS1* mRNA expression in women can be explained by hormonal factors.

## 5. Conclusions

In conclusion,our results evidence that both the AnxA1 and Gal-1 anti-inflammatory proteins are overexpressed in precancerous gastric lesions such as intestinal metaplasia and gastric ulcer. These results, together with the data of our previous study in chronic gastritis and gastric cancer [46], allow suggesting their involvement in gastric carcinogenesis from the early stages through the tumor progression cascade, possibly due to the inflammatory process of the gastric mucosa. However, the deregulated expression occurs regardless of the *H. pylori* infection and *cagA *virulence genotype. Further investigations are necessary to clarify the mechanisms of action of these proteins in the progression from precancerous lesions to cancer.

## Figures and Tables

**Table 1:** Sequence of primers and size of the fragments generated to determine *H. pylori *infection and *cagA *genotype.

Gene	Position	GenBank* access	Sequence 5′-3′	Fragment size (bp)
*CYP1A1 *	45844322 45844547	NW_004078084.1	F: TTTGGAAGTGCTCACAGCAG R: CTCACCCCTGATGGTGCTAT	226
*UreA *	754333 754648	CP006610.1	F: TTCCTGATGGGACCAAACTC R: TTACCGCCAATGTCAATCAA	316
*tsaA *	37 449	AY762757.1	F: CCTGCCGTTTTAGGAAACAA R: TCCGCATTCCTACCTAATGG	413
*cagA *	1 244	JF798698.1	F: TGACTAACGAAACTATTGATC R: CAGGATTTTTGATCGCTTTATT	244

*http://www.ncbi.nlm.nih.gov/genbank/.

**Table 2:** Relative gene expression of *ANXA1 *and *LGALS1* mRNA in the intestinal metaplasia (IM) and gastric ulcer (GU) groups and comparison according to risk factors.

Variables	*ANXA1 *				*LGALS1 *			
	IM		GU		IM		GU	
	(*N* = 36)		(*N* = 29)		(*N* = 36)		(*N* = 29)	
mRNA								
Mean ± SD	6.22 ± 1.43		6.69 ± 1.56		0.35 ± 1.37		0.69 ± 1.65	
(range)	(2.55–8.90)		(2.42–8.80)		(−3.54–2.86)		(−4.04–3.13)	
*P*	0.21				0.38			
Age (years)								
	<60	16 (44.5%)	<55	15 (51.7%)	<60	16 (44.5%)	<55	15 (51.7%)
(Mean ± SD)		6.48 ± 1.55		6.81 ± 1.71		0.62 ± 1.11		0.63 ± 1.84
	≥60	20 (55.5%)	≥55	14 (48.3%)	≥60	20 (55.5%)	≥55	14 (48.3%)
(Mean ± SD)		6.00 ± 1.32		6.55 ± 1.43		0.14 ± 1.53		0.76 ± 1.50
*P*	0.34		0.66		0.28		0.83	
Gender								
Female	19 (52.8%)		8 (27.6%)		19 (52.8%)		8 (27.6%)	
(Mean ± SD)	6.30 ± 1.34		7.07 ± 1.18		0.80 ± 1.30		1.41 ± 1.26	
Male	17 (47.2%)		21 (72.4%)		17 (47.2%)		21 (72.4%)	
(Mean ± SD)	6.12 ± 1.55		6.54 ± 1.69		−0.15 ± 1.29		0.42 ± 1.73	
*P*	0.71		0.35		0.03*		0.11	
Drinking								
Yes	10 (27.8%)		11 (39.3%)		10 (27.8%)		11 (39.3%)	
(Mean ± SD)	6.12 ± 1.26		6.17 ± 1.97		−0.13 ± 1.75		0.29 ± 1.81	
No	26 (72.2%)		17 (60.7%)		26 (72.2%)		17 (60.7%)	
(Mean ± SD)	6.26 ± 1.51		7.10 ± 1.17		0.54 ± 1.17		0.88 ± 1.58	
*P*	0.78		0.18		0.29		0.39	
Smoking								
Yes	26 (72.2%)		18 (64.3%)		26 (72.2%)		18 (64.3%)	
(Mean ± SD)	6.26 ± 1.49		6.66 ± 1.64		0.29 ± 1.49		0.46 ± 1.88	
No	10 (27.8%)		10 (35.7%)		10 (27.8%)		10 (35.7%)	
(Mean ± SD)	6.11 ± 1.32		6.86 ± 1.52		0.53 ± 1.01		0.98 ± 1.21	
*P*	0.76		0.75		0.58		0.39	
*H. pylori *								
Positive	14 (38.9%)		15 (55.5%)		14 (38.9%)		15 (55.5%)	
(Mean ± SD)	6.70 ± 1.75		6.88 ± 1.35		0.29 ± 1.61		0.94 ± 1.59	
Negative	22 (61.1%)		12 (44.5%)		22 (61.1%)		12 (44.5%)	
(Mean ± SD)	5.91 ± 1.11		6.47 ± 1.86		0.39 ± 1.23		0.67 ± 1.77	
*P*	0.15		0.53		0.85		0.69	
Genotype *cagA* ^a^								
Positive	12 (85.7%)		10 (66.7%)		12 (85.7%)		10 (66.7%)	
(Mean ± SD)	6.88 ± 1.81		6.82 ± 1.60		0.45 ± 1.58		0.50 ± 1.68	
Negative	2 (14.3%)		5 (33.3%)		2 (14.3%)		5 (33.3%)	
(Mean ± SD)	5.58 ± 0.96		6.99 ± 0.81		−0.62 ± 2.12		1.81 ± 1.03	
*P*	0.20		0.77		0.55		0.16	

^a^Nonparametric Mann-Whitney test; *significant difference.
